# Universal health coverage and the poor: to what extent are health financing policies making a difference? Evidence from a benefit incidence analysis in Zambia

**DOI:** 10.1186/s12889-022-13923-1

**Published:** 2022-08-13

**Authors:** Martin Rudasingwa, Manuela De Allegri, Chrispin Mphuka, Collins Chansa, Edmund Yeboah, Emmanuel Bonnet, Valéry Ridde, Bona Mukosha Chitah

**Affiliations:** 1 Heidelberg Institute of Global Health, University Hospital & Medical Faculty, Heidelberg University, Heidelberg, Germany; 2 Department of Economics, University of Zambia, Lusaka, Zambia; 3 IRD, UMR 215 Prodig, CNRS, Université Paris 1 Panthéon-Sorbonne, AgroParisTech, 5, Cours des Humanités, F-93 322 Aubervilliers Cedex, Paris, France; 4 CEPED, Institute for Research on Sustainable Development, IRD-Université de Paris, ERL INSERM SAGESUD, Paris, France

**Keywords:** UHC, Health financing, Benefit incidence analysis, Health benefits, Zambia

## Abstract

**Background:**

Zambia has invested in several healthcare financing reforms aimed at achieving universal access to health services. Several evaluations have investigated the effects of these reforms on the utilization of health services. However, only one study has assessed the distributional incidence of health spending across different socioeconomic groups, but without differentiating between public and overall health spending and between curative and maternal health services. Our study aims to fill this gap by undertaking a quasi-longitudinal benefit incidence analysis of public and overall health spending between 2006 and 2014.

**Methods:**

We conducted a Benefit Incidence Analysis (BIA) to measure the socioeconomic inequality of public and overall health spending on curative services and institutional delivery across different health facility typologies at three time points. We combined data from household surveys and National Health Accounts.

**Results:**

Results showed that public (concentration index of − 0.003; SE 0.027 in 2006 and − 0.207; SE 0.011 in 2014) and overall (0.050; SE 0.033 in 2006 and − 0.169; SE 0.011 in 2014) health spending on curative services tended to benefit the poorer segments of the population while public (0.241; SE 0.018 in 2007 and 0.120; SE 0.007 in 2014) and overall health spending (0.051; SE 0.022 in 2007 and 0.116; SE 0.007 in 2014) on institutional delivery tended to benefit the least-poor. Higher inequalities were observed at higher care levels for both curative and institutional delivery services.

**Conclusion:**

Our findings suggest that the implementation of UHC policies in Zambia led to a reduction in socioeconomic inequality in health spending, particularly at health centres and for curative care. Further action is needed to address existing barriers for the poor to benefit from health spending on curative services and at higher levels of care.

## Introduction

Following the global call to reduce persistent inequalities in health and access to health services, various health reforms designed towards the attainment of Universal Health Coverage (UHC) have been implemented in several countries, especially in Sub-Saharan Africa [1–4]. One of the UHC principles involves ensuring that access and utilization of health services ought to be based on the need for care and not on ability to pay [5]. In other words, the ultimate goal of UHC is to reduce or eliminate the inequalities in benefiting from investments in health policies [6]. Therefore, understanding the distribution of health benefits from UHC-reforms among different socioeconomic groups represents a relevant health policy question, which health systems should address to ensure access to and utilization of health services among the vulnerable and poor population [7].

While all countries, rich and poor, aspire to achieve universal access to needed and good quality health services, low and middle-income countries (LMICs) are lagging behind in this endeavour. LMICs have taken different paths to achieve UHC and have invested in different UHC reforms, such as social health insurance schemes, user fee removal, voucher schemes, and results-based financing [8]. Despite these large investments, inequalities in access and utilization of health services in LMICs still exist. This raises questions on the ability of UHC reforms to facilitate change towards equitable financing, access and utilization of healthcare benefits in these countries. As observed by Wagstaff et al. [7] and Yaya & Ghose [9], the aforementioned inequalities can be caused by various factors including medical and non-medical costs associated with using healthcare, geographical deprivations and contextual barriers.

As investments towards UHC continue to grow, it is important to ensure that no one is left behind and that the investments made contribute to closing existing gaps in access, health spending, and health rather than contributing to widening them [10, 11]. Evidence of the effects of the specific UHC-reforms on access to and utilization of health services is growing. Various studies have indicated positive effects of UHC-reforms in reducing health inequalities in LMICs, but the least-poor still enjoy more health benefits than the poor segments of the population [2, 3, 7, 12, 13]. Therefore, LMICs are determined to increase their investments towards more equitable health systems by removing all barriers that are still hindering the poor segments of the population from accessing needed healthcare. Yet, evidence on whether the investments made to foster UHC have benefitted poor segments of the population is still insufficient. Understanding the extent to which health benefits are distributed across different socioeconomic groups would inform effective allocation of financial resources based on the need for health services. A few studies have relied on Benefit incidence analysis (BIA) to assess the distributional incidence of health spending in LMICs and indicated mixed distributional patterns dominated by a pro-rich bias in health spending [7, 14–16] **.** Most of these BIA studies have been conducted at one point in time without allowing the assessment of changes in distributional incidence of health spending over time or examining the relationship to the implementation of specific policy reforms. Additionally, most prior BIA studies have focused on assessing the distributional incidence of public spending, ignoring donor and private spending, which make up a substantial share of the total health expenditures in many LMICs [14, 17, 18]**.**

In the last three decades, Zambia has implemented an array of UHC-reforms to increase access and utilization of health services among all socioeconomic groups of the population [19]. These includes: decentralization of health services planning and delivery; nationwide performance-based contracting (PBC); introduction and subsequent abolition of user fees in rural areas, peri-urban areas, and all primary health care facilities nationwide [14, 15] development and application of a needs-based formula for allocating operational grants from the Ministry of Health headquarters to the districts; discontinuation of PBC and introduction of results-based financing (RBF) in 11 districts with a focus on maternal and child health [16]. These reforms are inclined towards maternal and child health, given that a large number of mothers and children are still dying in Zambia despite significant reductions in maternal and child mortality over the past two decades. By the end of 2018, the maternal mortality ratio and under-five mortality rate were estimated at 252 deaths per 100,000 live births and 61 deaths per 1000 live births, respectively [17]. These results are above the average for lower- middle-income countries which means that Zambia is worse off. Despite the adoption of several health reforms in Zambia, there is insufficient evidence on their effects on facilitating equity of access to quality healthcare. For instance, studies that have looked at the effect of removing user fees in Zambia show that socio-economic and geographical disparities in out-of-pocket expenditure (OOPE) and access to healthcare still exist [20]. Further, two studies found that about 11% of all households seeking healthcare had to borrow a substantial amount of money or sell valuable assets to pay for healthcare [21, 22] and also found no evidence that removal of user fees in Zambia has increased health care utilization among the poorest group at national level. Only a few studies indicated increased utilization of health services associated with user fee abolition. Two studies have indicated an increase in primary health services utilization in rural areas [23, 24]. The percentage of institutional deliveries increased from 44% in 2002 to 84% in 2018 [25] and two studies found an increase of institutional deliveries associated with removal of user fees [26, 27]. According to the latest available data on utilization of curative healthcare services, the per annum per capita utilization rate among the lowest and the highest quintile groups was estimated at 1.9 and 1.4, respectively [28]. Regarding PBC, a study by Chansa et al. [29] concludes that PBC is a cost-efficient and sustainable policy reform, and it can contribute to improved equity of access to maternal health services. Lastly, on RBF, a study by Zeng et al. [30] has shown that RBF and input-based financing were cost-effective in Zambia. Nonetheless, Paul et al. [31] suggest that providing more resources to health facilities may be more effective in the Zambian context of free care at the entire primary care level than RBF from an efficiency point of view.

Very few studies in Zambia have looked at the distributional incidence of health spending in line of the implemented UHC-reforms. A recent BIA study by Chitah et al. [19] observes that there has been a pro-poor redistribution of health benefits but health benefits being received by the poor are still lower than their health needs. However, the study by Chitah et al. [19] only focused on the distributional incidence of public spending rather than the overall spending (i.e., public, donor, and out-of-pocket expenditure) in the health sector. Secondly, there was no stratification of the analysis by programmatic areas such as curative care and maternal health despite the inclination of UHC policy reforms in Zambia towards diseases and conditions with the highest burden, particularly maternal health.

**Fig. 1:**
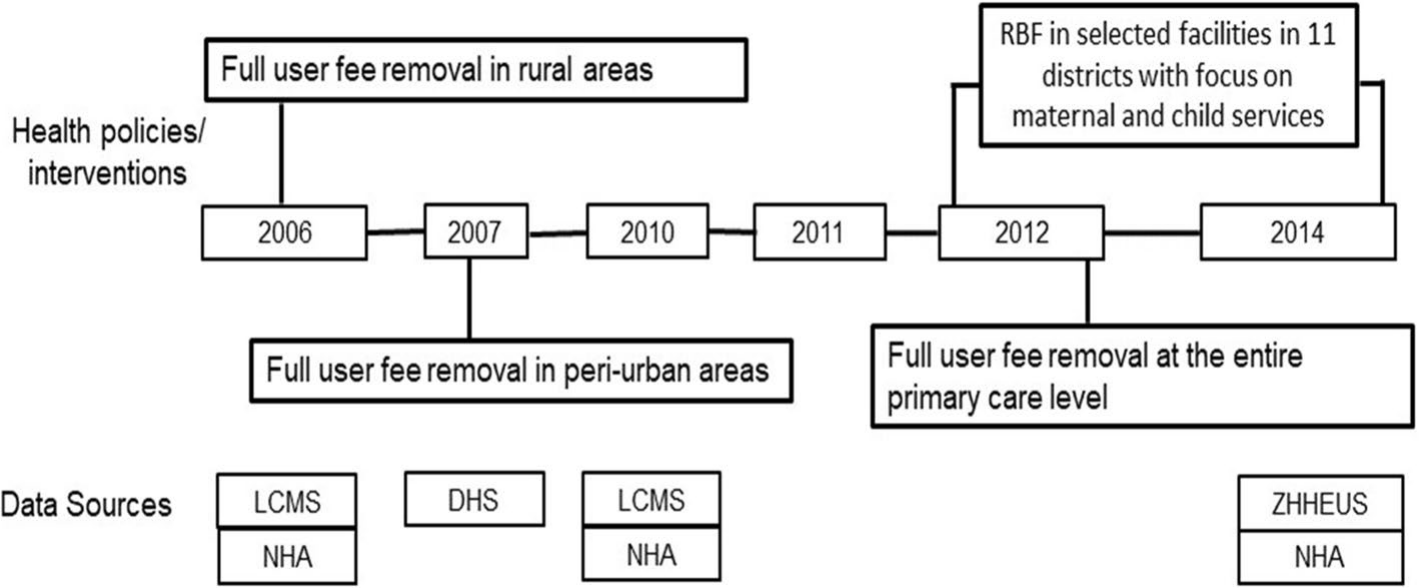
Timeline of health policies and interventions targeting curative and maternal services

Our study aims to fill this knowledge gap by assessing changes over time in the distributional incidence of public and overall health spending on curative services and institutional delivery (childbirth at a health facility) in Zambia. As depicted in the Fig. 1, the analysis was undertaken at three time points – 2006/7, 2010 and 2014 – to assess changes in the distributional incidence of health spending in line with the UHC reforms in the country. Looking at overall spending on health is critically important because in Zambia (just like several other developing countries), public spending on health is less than 50% of the total health expenditure. According to the Ministry of Health [32], government expenditure as a share of the total health expenditure was about 41% on average over the period 2013–2016.

## Methods

### Study design

We applied BIA to assess the distributional incidence of both public and overall health spending on curative services and institutional delivery at three time points. BIA measures the share of benefits accruing to different socioeconomic groups from using health services at a specific point in time, thereby determining whether financial health benefits are reaching the poor segments of the population ([18, 33]. BIA relies on two sets of data: health service utilization stratified by socioeconomic status and recurrent health spending on different types of health services. In other words, BIA expresses in monetary terms the distribution of health benefits. We performed a quasi-longitudinal analysis using data from available nationally representative repeated cross-sectional household surveys and national health accounts (NHA) for the health service utilization and health spending, respectively. Before deciding on the time points of our analysis, we mapped all the health policies and interventions (Fig. 1) that were implemented in Zambia with the aim of achieving universal coverage of curative and maternal health services. Based on the available data, we then chose the time points that could allow us to assess the changes of socioeconomic inequality in financial health benefits over time in line with the implemented UHC-reforms.

### Data sources and measurement of health service utilization

We derived data on healthcare utilization from the 2006 and 2010 Living Condition and Monitoring surveys (LCMS) and the 2014 Zambia Household Health Expenditure and Utilization Survey (ZHHEUS) for the curative services and the 2007 Demographic and Health Surveys (DHS) and the 2014 ZHHEUS for institutional delivery. As summarized in Table 1, these household surveys are nationally representative and contain data on the utilization of curative services and institutional deliveries differentiated by provider typology and socioeconomic status (SES). The latter allowed us to group individuals into weighted SES quintiles, from the poorest to the least poor. Table 2 indicates the health variables we extracted from each household survey. Given data availability, we relied on different data to compute household SES, the basis for our classification of individuals into groups. For analyses relying on LCMS and ZHHEUS, we used the per capita consumption expenditure based on the total household food and non-food expenditure. For analyses relying on DHS, we used the household-wealth-index factor scores generated through the principal component analysis based on the household material asset ownership from the DHS.

**Table 1:** Summary information on population survey data employed in the study

Health service utilization indicator	Household survey	Year	When the survey was conducted	Sampling strategies
Use of curative services by level of care and stratified by socio-economic status	Living Condition and Monitoring surveys (LCMS)	2006	January–December 2006	Stratified two-stage sampling technique: In the first stage, the primary units or enumeration areas (EAs) were drawn to probability proportional to the number of households counted in the EA (for a total of approximately 1000 EAs). In the second stage, households were drawn in equal probability in each of the enumeration areas (for a total of approximately 20,000 households).
		2010	January–April 2010	
Use of institutional delivery by level of care and stratified by socio-economic status	Demographic and Health Survey (DHS)	2007	April – October 2007	Stratified two-stage sampling technique: In the first stage, 320 EAs were selected with probability proportional to the SEA size. An EA is a convenient geographical area with an average size of 130 households or 600 people. In the second stage, households were drawn with equal probability in each EA (for a total of approximately 8000 households).
Use of curative services and institutional deliveries by level of care and stratified by socio-economic status	Zambia Household Health Expenditure and Utilization Survey (ZHHEUS)	2014	January to April, 2014	A two-stage stratified cluster sample in the first stage, 320 EAs were selected within each stratum using the probability proportional to estimated size procedure. During the second stage, 20 households were selected from each EA using the systematic random sampling method. A total of 14,000 households were sampled and interviewed with replacements.

**Table 2:** Variables and data sources

Variables and data sources	Healthcare providers	Data sources (years)	NHA data (year)	Sources for OOPE unit cost adjustment
Curative health service utilization for adults and children in the prior two weeks	Public health centres, public district hospitals, public tertiary hospitals, mission facilities, private facilities	LCMS (2006; 2010) ZHH EUS (2014)	2006 2010 2014	ZHHEUS 2014
Institutional deliveries	Public hospitals, public health centres, mission hospitals, mission health centres, and private facilities	DHS (2007) ZHHEUS (2014)	2006 2014	ZHHEUS 2014

To estimate the annual visits for curative healthcare services and institutional deliveries, we adopt the methodological guidance provided by McIntyre and Ataguba [18] For curative services, we used a binary variable indicating whether the individuals used curative services in the previous 14 days and for the institutional delivery, we used a binary variable indicating whether the women delivered in the study year. Curative care visits were annualized to obtain visits per year by multiplying the visits in a recall period of 14 days by 26. We categorized curative services and institutional delivery by different providers and types of health facilities depending on data availability in each survey and NHA.

### Measurement of health expenditures and unit costs

We derived data on health spending from the NHA. We estimated the unit cost of curative health services and institutional deliveries using recurrent public spending, donor spending and household OOPE from the NHA. We applied a constant unit subsidy assumption to estimate the unity subsidy for public and donor spending at different providers/types of health facilities. For the OOPE, we relied on a constant unit cost for each quintile based on the percentage of OOPE incurred by each quintile at different providers/types of health facilities. The OOPE adjustment was made because individuals belonging to different SES quintiles have different abilities to pay for OOPE at different providers/types of facilities. Hence using a constant unit OOPE at each provider/type of facility would overestimate the OOPE incurred by the bottom SES quintiles. We used the data on household health expenditure from the ZHHEUS survey to quantify the distribution of OOPE on health across socioeconomic quintiles. To determine the unit subsidy or the unit cost at each provider/type of health facility, we divided the total health spending by the total utilization of health services at each health facility.

### Analytical approach

We computed the traditional BIA by measuring the distributional incidence of public spending and comprehensive BIA by looking at the distributional incidence of overall health spending, including public and donor subsidies allocated to different health facilities and OOPE incurred by individuals. We repeated the same analysis at three time points for the curative services and at two time points for institutional delivery to capture changes in the distribution of health spending over time. Based on data availability (Table 2), we stratified our analysis by health facility typologies (public health centres, public hospitals and mission health facilities) for each year. Given the limited number of private health facilities in Zambia, they were excluded from the analysis. To determine the total financial health benefits at each provider/type of health facility, we multiplied the unit subsidy or unit cost by the total utilization of health services at each provider/type of health facility. We used concentration indices to measure the degree of inequality in the distribution of public and overall health spending on curative services and institutional delivery across different socioeconomic groups. The concentration index (CI) quantifies the degree of wealth-related inequality and ranges from − 1.0 to + 1.0. The CI takes a negative (positive) value when the financial health benefits is concentrated among the poor (least-poor). If the CI is close to zero, a lower degree of inequality is present; and if it is zero, there is no wealth-related inequality [33].

The standardized concentration index (*C*_*h*_) is estimated as follows [33]:

$${C}_h=\frac{2 Cov\ \left({h}_i,{R}_i\right)}{\mu }$$

Where *h*_*i*_ is the health variable (e.g. healthcare utilization) for individual ί, μ is the mean of health variable, *R*_*i*_ is individual i’s fraction socioeconomic rank, and *Cov* (*h*_*i*_, *R*_*i*_) is the covariance. We used convenient regression ([34] to allow the calculation of the standard errors of the concentration index. The formula is:

$$2\sigma {\displaystyle \begin{array}{c}2\\ {}R\end{array}}\left[\frac{h_i}{\mu}\right]=\alpha +{\beta R}_i+{\varepsilon}_i$$

Where $$2\sigma {\displaystyle \begin{array}{c}2\\ {}R\end{array}}$$ is the variance of the fractional rank variable. *β* is the estimator of the concentration index.

## Results

### Benefit incidence of public spending on curative health services

The results in Table 3 show that total public spending on curative health services was generally pro-poor during the period under review and increased steadily from a CI of − 0.003 in 2006 to − 0.207 in 2014. However, there is a difference when public spending on curative health services is stratified by provider/type of health facility. Public health spending on curative health services at public health centres and mission health facilities tended to be pro-poor but least-poor at public hospitals. The distributional incidence of public spending on curative health services at public health centres was near equality in 2006 (CI = 0.025) but shifted to a pro-poor distribution in 2010 (CI = − 0.033) and increased to a CI of − 0.163 in 2014. Public health spending on curative health services at mission health facilities was pro-poor with the CI increasing from − 0.081 in 2006 to a CI of − 0.225 in 2014. On the other hand, public health spending at public hospitals stayed in favour of the least-poor segments of the population throughout the period under review. The CI at public hospitals increased from 0.083 in 2006 to 0.207 in 2014 in favour of the least-poor.

**Table 3:** Benefit incidence of public spending on curative health services

Year	2006	2010	2014	Difference 2010–2006	Difference 2014–2010	Difference 2014–2006
Health care provider/Facility type	CI (SE)	CI (SE)	CI (SE)	CI (SE)	CI (SE)	CI (SE)
All public and mission health facilities	− 0.003 (0.027)	− 0.049*** (0.005)	− 0.207*** (0.011)	− 0.045* (0.027)	−0.158*** (0.012)	− 0.203*** (0.011)
Public health centres	0.025 (0.042)	−0.033* (0.019)	−0.163*** (0.014)	− 0.058 (0.046)	−0.129*** (0.0233)	− 0.187*** (0.038)
Public hospitals	0.083*** (0.028)	0.092*** (0.023)	0.207*** (0.015)	0.009 (0.037)	0.115*** (0.041)	0.124*** (0.038)
Mission health facilities	−0.081 (0.066)	−0.022 (0.076)	− 0.225*** (0.059)	−0.059 (0.101)	− 0.203** (0.090)	−0.144** (0.075)

*CI* Concentration index; *SE* Standard error; Statistically significant: ***p < 0.01; **p < 0.05; *p < 0.1

### Benefit incidence of overall spending on curative health services

Overall health spending on curative services (Table 4) was in favour of the least-poor in 2006 (CI = 0.050), but became pro-poor in 2010 (CI = − 0.030); and further increased to a CI of − 0.169 in 2014. When overall health spending on curative services is stratified by provider/type of health facility, the distribution pattern remains pro-poor for all types of health facilities except for public hospitals in 2006 and 2010. In 2014 the distribution was pro-poor for public hospitals but the result is statistically insignificant. Overall health spending on curative services at public health centres and mission health facilities was pro-poor for all the years.

**Table 4:** Benefit incidence analysis of overall health spending on curative health services

Year	2006	2010	2014	Difference 2010–2006	Difference 2014–2010	Difference 2014–2006
Health care provider/Facility type	CI (SE)	CI (SE)	CI (SE)	CI (SE)	CI (SE)	CI (SE)
All public and mission health facilities	0.050 (0.033)	−0.030*** (0.003)	−0.169*** (0.011)	− 0.080** (0.033)	−0.139*** (0.011)	− 0.220*** (0.031)
Public health centres	−0.003 (0.036)	− 0.056*** (0.014)	−0.135*** (0.010)	− 0.062 (0.041)	0.079*** (0.018)	− 0.141*** (0.035)
Public hospitals	0.069** (0.029)	0.085*** (0.022)	−0.066 (0.048)	−0.011 (0.036)	− 0.152*** (0.052)	−0.140*** (0.052)
Mission health facilities	−0.081 (0.065)	−0.088 (0.058)	− 0.216** (0.066)	−0.007 (0.067)	− 0.128* (0.085)	−0.136* (0.079)

*CI* Concentration index; *SE* Standard error; Statistically significant: ***p < 0.01; **p < 0.05; *p < 0.1

### Benefit incidence of public spending on institutional delivery

Total public health spending on institutional deliveries mostly benefited the least-poor women over time even though the CI reduced from 0.241 in 2007 to 0.120 in 2014 (Table 5). Stratified results show the same pattern at public hospitals with the CI declining slightly from 0.340 in 2007 to 0.304 in 2014. Public spending on institutional deliveries at public health centres mostly benefited the least-poor in 2007 (CI = 0.181) but this changed in 2014 when the distribution became pro-poor (CI = − 0.037). A different picture is observed for public spending on institutional deliveries at mission health facilities which stayed pro-poor for all the years. However, the CI decreased from − 0.217 in 2007 to − 0.070 in 2014.

**Table 5:** Benefit incidence of public health spending on institutional deliveries

Year	2007	2014	Difference 2014–2007
Health care provider/Facility type	CI (SE)	CI (SE)	CI (SE)
All public and mission health facilities	0.241*** (0.018)	0.120*** (0.007)	−0.121*** (0.019)
Public Hospitals	0.340** (0.03)	0.304** (0.022)	−0.035* (0.041)
Public health centres	0.181** (0.028)	−0.037** (0.003)	−0.219** (0.028)
Mission health facilities	−0.217** (0.070)	−0.070** (0.054)	0.147** (0.088)

*CI* Concentration index; *SE* Standard error; Statistically significant: ***p < 0.01; **p < 0.05; *p < 0.1

### Benefit incidence of overall spending on institutional delivery

Overall health spending on institutional deliveries (Table 6) favoured the least-poor women throughout the period under review with the CI increasing from 0.051 in 2007 to 0.116 in 2014. The same pattern was observed at public hospitals with the CI increasing from 0.054 in 2007 to 0.291 in 2014. At both public health centres and mission health facilities, overall health spending on institutional deliveries favoured the least-poor in 2007 but this changed in 2014 when the distributions became pro-poor.

**Table 6:** Benefit incidence analysis of overall health spending on institutional deliveries

Year	2007	2014	Difference 2014–2007
Health care provider/Facility type	CI (SE)	CI (SE)	CI (SE)
All public and mission health facilities	0.051** (0.022)	0.116*** (0.007)	0.066** (0.023)
Public hospitals	0.054** (0.036)	0.291** (0.022)	0.054* (0.036)
Public health centres	0.050* (0.027)	−0.029** (0.003)	−0.079** (0.027)
Mission health facilities	0.046** (0.101)	−0.066** (0.054)	−0.112* (0.115)

*CI* Concentration index; *SE* Standard error; Statistically significant: ***p < 0.01; **p < 0.05; *p < 0.1

## Discussion

This study sought to examine changes in the distribution of public and overall health spending (public, donor, and OOPE) for curative services and institutional deliveries as UHC reforms were being implemented in Zambia. The study makes an important contribution to the literature on UHC, being the first to assess the changes in the distributional incidence of public and overall health spending over time and also differentiating between curative and maternal care services in Zambia. Given the complexity of attributing change to individual UHC policies, and the data available, our study falls short of being able to attribute the distributional patterns to any specific UHC reform, but nonetheless examines changes overtime in relation to these reforms. Overall, we observe that public and overall health spending on curative services tended to benefit the poorer segments of the population while public and overall health spending on institutional delivery tended to benefit the least-poor. For both curative services and institutional deliveries, health spending at higher levels of health care (public hospitals) benefited the least-poor more than the poor while at lower levels of health care (health centres) and mission health facilities, the poor benefited more.

Zambia removed user fees in all rural areas in 2006, in peri-urban areas in 2007, and across the entire primary health care level in 2012 [20, 24] to address inequalities in access and utilization of health services. Three systematic reviews on user fees removal in LMICs by Qin et al. [35], Dzakpasu et al. [36], and Lagarde & Palmer [37] suggest that removing user fees has the potential to increase the utilization of both curative and maternal health services, especially for the poor. Our findings are consistent with results from previous studies in Zambia [20, 23, 24] which revealed that the removal of user fees in Zambia has contributed to increased utilization of curative services by the poor in Zambia. Public and overall spending on curative services benefited more the poor than the least-poor overtime. Given that most of the public health facilities providing primary health care are located in rural areas where the majority of the poor live and where about 90% of patients seek care in public facilities [38]; the removal of user fees has contributed to increased utilization of curative services among the poor. This pro-poor distribution of benefits from health spending on curative services is positively surprising, considering that Zambia has not adopted any specific policy to protect the ultra-poor from informal payments for healthcare. This evidence is inconsistent with evidence from Malawi, a neighbour country of Zambia, which has never introduced user fees but has high OOPE associated with using curative services that hinder the poorer segments of the population from using curative services ([39, 40]. For Zambia, Masiye and colleagues [41] observe that patients incur informal payments for health services that should be offered at free of charge. This presents a financial barrier for the poor segments of the population to use formal care [22]. The inequality on curative healthcare services is likely partly mitigated by the elimination of user fees with the effect on inequality reduction across the board. The share of donor funding in overall spending further enhances the equality aspects due to the focus on primary care. Contrary to curative services, our findings on institutional delivery reveal that the overall distributional incidence for the relevant public and overall health spending is in favour of the least-poor. These results are consistent with findings by Chama-Chiliba & Koch [42] who conclude that removal of user fees has not fully removed barriers to utilisation of delivery services at public facilities in Zambia. Findings from Burkina Faso also question the fidelity of the free care policy in Zambia in ensuring free access to institutional deliveries [43]. A study by Sochas [44] further reveals that health facility rules in Zambia can influence women’s behaviour during pregnancy and childbirth, and create inequities against women with fewer financial resources. As part of the rules, pregnant women are required to purchase items needed for the delivery at a health facility such as bleach, a bathing tub, bucket, plastic sheet, gloves, nappies, and cotton wrapper, among others. In addition, costs for transport and new clothes for the babies and mothers are incurred (Scott et al., 2018). Consequently, inability to cater for costs associated with childbirth leads to low institutional deliveries in Zambia, especially for women from poor households [45]. Kaonga and colleagues [22] also show that female-headed households bear the highest financial burden of healthcare payments in Zambia. This suggests that the costs associated with seeking care are still an important barrier to institutional deliveries among poor women in Zambia. The decrease of the inequality in public and overall spending on institutional deliveries between 2007 and 2014 implies that the removal of user fees may have had a positive effect, but was not fully effective in removing all the financial burden among poor women who would wish to deliver at a health facility [43]. Other than affordability and as observed in other LMICs [46, 47], there are other dimensions of the health system environment in Zambia such as geographical accessibility, cultural beliefs, availability, and perceived quality of care that can negatively affect institutional deliveries [48]. Therefore, to eliminate the inequality in the distribution of health spending on institutional deliveries, the Zambian government needs to implement strategies aimed at removing financial and non-financial barriers associated with childbirth at a health facility, especially for the poor segments of the population.

Consistent with previous studies in LMICs [14, 19, 49, 50], inequalities in health spending on both curative services and institutional deliveries remain high for higher levels of care (i.e., inpatient care and deliveries at hospitals). This implies that UHC policies are not very effective at public hospitals. This could be because the user fee removal policy in Zambia is only applicable at lower levels of the public healthcare delivery system. In line with a study from India [51] and Zambia [19]; our findings indicate that health spending for both curative services and institutional deliveries at public health centres and mission health facilities, which operate at a lower level of healthcare and mostly in rural areas, tended to become more pro-poor over time likely due to the user fee removal policy. It should be emphasised that we observe a greater effect in increased equity in health facilities mostly located in rural (e.g. health centres and mission health facilities) compared to health facilities mostly located in urban (e.g. hospitals) areas, probably due to the fact that user fee removal was first introduced in rural (2006) and then in urban (2010) settlements. The performance-based financing scheme, which was implemented between 2012 and 2014 at public health centres in some districts with a focus on maternal and child services—could have also contributed to greater equality of health benefits at the lower level of healthcare provision [30, 52]. Contrary to lower level of healthcare, individuals who access hospital services directly incur bypass fees or pay to access high-cost schemes and hospital prepayment medical schemes which are unaffordable to the poor. Except for emergency cases, a bypass fee is charged to patients who present themselves for treatment at a hospital without being referred from a health centre. Individuals from richer households can afford to pay the bypass fee and register for hospital prepayment schemes but this is not the case with poorer households. The existence of these charges at public hospitals in Zambia could explain why there are still disparities in the financing and utilization of healthcare services in Zambia [20]. The other reason public and overall health spending favour the least-poor at public hospitals is that most of the tertiary and general hospitals are located in urban areas while the majority of the poor segments of the population live in rural areas where there are mostly public health centres and mission health facilities. As observed by Hjortsburg [53] and Eckman [54], the cost of providing health care in Zambia is skewed towards the urban areas, while access and consequences are concentrated among the rural areas and poorer socio-economic groups. Furthermore, there is an erratic supply of delivery kits, drugs, and other medical supplies at public hospitals as compared to public health centres [55]. The scarcity of healthcare resources presents a high financial burden for the poor at higher levels of healthcare [41, 56]. As the core goal of UHC is that all people get access to needed high-quality healthcare regardless of one’s ability to pay [5], our findings call for specific actions by the Zambian Government to lift the financial and non-financial barriers that are still hindering the poor from using services at higher level of the healthcare delivery system. Such actions may be targeted towards some of the following areas: improving the referral system; improving the distribution and availability of human resources particularly addressing the imbalance between the rural and urban areas; improving and ensuring the drug stock availability for essential medicines; improving the availability of diagnostic services (e.g. laboratory and x-ray services); formulating and adhering to a transparent priority setting process and related resource allocation process that assists in addressing the skewed imbalances in health care resources and to some extent health status outcomes.

### Methodological considerations

Notwithstanding the value of this study, we need to note some limitations. Firstly, LCMS, DHS and ZHHEUS household surveys classify individuals across socioeconomic groups differently. Therefore, the socioeconomic groups may not be fully comparable across these surveys and we need to acknowledge bias that may arise from the use of different socioeconomic status measures. Secondly, based on the data at our disposal, having applied the constant unit subsidy/cost assumption, we might have masked differences in financial health benefits accruing to people of different socioeconomic groups at different health facilities or in different geographical settings. Thirdly, this study focused on the distribution of benefits from using curative services and institutional deliveries, expressed in monetary terms, without looking at health need and healthcare quality. Therefore, even if curative care and institutional deliveries were pro-poor at both public health centres and mission health facilities, it is difficult to tell if the services which the clients received were of high quality. Further analysis taking into consideration the health needs, quality and demand for healthcare could be undertaken.

## Conclusion

The study concludes that the overall distributional incidence for both public and overall spending on health is pro-poor for curative services, but least-poor for institutional deliveries. Stratifying the analysis by provider/type of health facility shows that for both curative services and institutional deliveries; health spending at public hospitals benefited the least-poor more than the poor while at public health centres and mission health facilities, the poor benefited more. This means that UHC policies in Zambia have likely translated into improved equity in health spending for curative services and institutional deliveries at health centres and mission health facilities but not at public hospitals. To address the problem of equity at higher levels of care highlighted by our analysis, there is need to put in place measures to facilitate access to public hospitals by the poor. This could be achieved by enrolling the poor and vulnerable in subsidized prepayment schemes, subsidizing direct payments for the poorer segment of the population at public hospitals and improving purchasing arrangements of health services.

## Data Availability

The original datasets from DHS (http://dhsprogram.com/) and LCMS (https://microdata.worldbank.org/index.php/catalog/lsms) are freely available. The original datasets from ZHHEUS and NHA are available from the corresponding author upon reasonable request.

## Abbreviations

BIA: Benefit Incidence Analysis
CI: Concentration Index
DHS: Demographic and Health Surveys
GDP: Gross Domestic Product
LCMS: Living Condition and Monitoring surveys
LMIC: Low-and-Middle Income Country
NHA: National Health Accounts
OOPE: Out-of-Pocket Expenditure
PBC: Performance-based Contracting
PBF: Performance-based Financing
UHC: Universal Health Coverage
ZHHEUS: Zambia Household Health Expenditure and Utilization Survey
